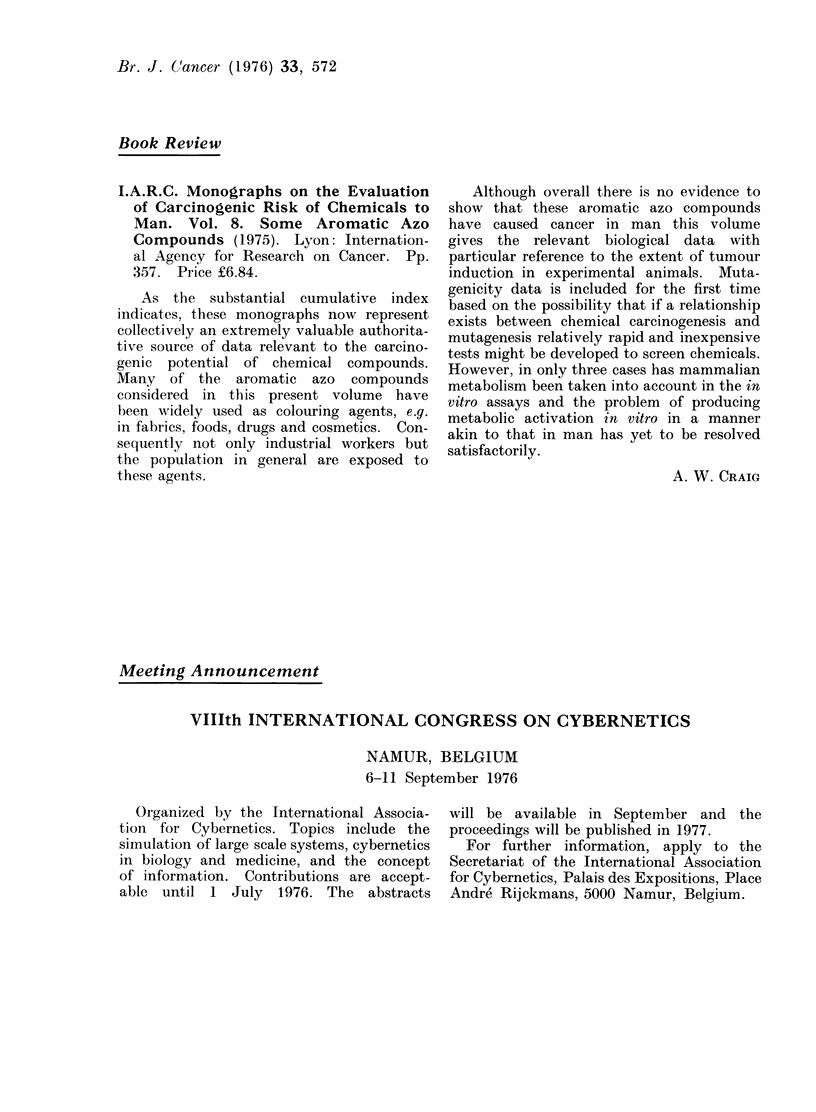# I.A.R.C. Monographs on the Evaluation of Carcinogenic Risk of Chemicals to Man Vol. 8. Some Aromatic Azo Compounds (1975)

**Published:** 1976-05

**Authors:** A. W. Craig


*Br. J. Cancer* (1976) **33**, 572

### *Book Review*

**I.A.R.C. Monographs on the Evaluation of Carcinogenic Risk of Chemicals to Man. Vol. 8. Some Aromatic Azo Compounds** (1975). Lyon: International Agency for Research on Cancer. Pp. 357. Price £6.84.

As the substantial cumulative index indicates, these monographs now represent collectively an extremely valuable authoritative source of data relevant to the carcinogenic potential of chemical compounds. Many of the aromatic azo compounds considered in this present volume have been widely used as colouring agents, *e.g.* in fabrics, foods, drugs and cosmetics. Consequently not only industrial workers but the population in general are exposed to these agents.

Although overall there is no evidence to show that these aromatic azo compounds have caused cancer in man this volume gives the relevant biological data with particular reference to the extent of tumour induction in experimental animals. Mutagenicity data is included for the first time based on the possibility that if a relationship exists between chemical carcinogenesis and mutagenesis relatively rapid and inexpensive tests might be developed to screen chemicals. However, in only three cases has mammalian metabolism been taken into account in the *in vitro* assays and the problem of producing metabolic activation *in vitro* in a manner akin to that in man has yet to be resolved satisfactorily.

A. W. CRAIG